# Management of Primary Obstructive Megaureter by Endoscopic High-Pressure Balloon Dilatation. IDEAL Framework Model as a New Tool for Systematic Review

**DOI:** 10.3389/fsurg.2019.00020

**Published:** 2019-04-16

**Authors:** Rosa M. Romero

**Affiliations:** Pediatric Urology Unit, Hospital Universitario Virgen del Rocío, Seville, Spain

**Keywords:** megaureter, endourologic treatment, research in surgery, evidence–based medicine, clinical outcome assessment (COA), CAKUT (congenital anomalies of the kidney and urinary tract), minimally invasive surgery (MIS)

## Abstract

**Introduction:** Therapeutic management of primary obstructive megaureter (POM) requiring surgery has been under debate for the last 15 years especially regarding the outcomes of endoscopic techniques compared to most traditional approaches. This review aims to analyze endoscopic High-Pressure Balloon Dilatation (HPBD) using the IDEAL model, a five-stage framework that describes surgical innovations (Idea, Development, Exploration, Assessment, and Long-term Study) and provides recommendations for a rigorous stepwise surgical research pathway. This model has been developed and demonstrated its value in evaluating surgical innovations assessing data quality and providing relevant information for the optimal design and feasibility of research in surgery.

**Materials and Methods:** A systematic review of the published series of endoscopic HPBD in patients with POM was done using the IDEAL model as a tool to assess evidence quality. Reported clinical outcomes are also analyzed and reviewed.

**Results:** The analysis of the results of the systematic assessment of the reported cohort of patients treated with HPBD for POM that the technique up to date is in stage 2a and stage 2b, or development. Evidence quality among the reported cohorts of patients with POM treated with HPBD is adequate, although systematization and standardization should be improved. Clinical outcomes of HPBD in the management of POM consistently show a 87.7% success rate with a negligible operative complication rate once “learning curve” has been surpassed. Symptomatic vesicoureteral reflux (VUR) is the main reason for ureteric reimplantation, but asymptomatic VUR does not seem to influence clinical outcome.

**Conclusions:** The IDEAL framework and recommendations have allowed a systematic analysis of the evidence quality of the reported experience in the management of children with POM with HPBD of the vesicoureteral junction. The available evidence demonstrates that HPBD is an effective treatment for patients with POM, with a long-term success rate of 87.7% with very low morbidity. Future research mandates a standardization of data reporting, “ideally” following IDEAL recommendations, that would be required for any intervention and facilitate comparative analysis.

## Introduction

The therapeutic approach of patients with primary obstructive megaureter POM that require surgery due to progressive dilatation, ongoing symptoms, and or renal function deterioration remains under debate. Although ureteral reimplantation with or without tapering has been acknowledged as the mainstream treatment for these patients, other approaches aiming to overcome the difficulties of reimplantation of a grossly dilated ureter into a small infant bladder and the potential morbidity associated with it have emerged (1).

There has been an increasing interest in a minimally invasive approach to this challenging issue, and different groups have reported a variety of endoscopic techniques.

The role of these different endoscopic techniques has not been well-established mainly due to the lack of consistent evidence to support it. Poor quality data or evidence is a generalized problem when defining the impact and potential safety implications of surgical innovations (2). This situation is especially aggravated in the case of the POM, given its relatively low incidence and the variability between working groups within pediatric urology (3).

Since first reported as a temporary treatment for complicated POM in 1998 by Angulo et al. endoscopic high pressure balloon dilatation (HPBD) of the vesicoureteral junction, has been used by different groups, evolved, and even changed the scope of its expected outcome from a temporary treatment to a definitive approach (4–6).

The evaluation of surgical or interventional techniques should be done with the appropriate model that takes into account both the technique and its development process, from the development of the concept to its modifications and expansion, to be able to adequately describe what their results are and compare them with other techniques (7, 8).

HPBD for POM is an endoscopic procedure that as other endourological procedures in children is a complex intervention, and its outcomes and therefore its assessment is affected by factors such as operator, team, equipment, setting, learning curves among others (9). These characteristics have led to persistent difficulties in obtaining high-quality evidence to determine the role of HPBD in the management of POM that requires surgical intervention.

The IDEAL framework provides an excellent tool in the complex task of evaluating a new surgical technique or procedure, even more complex in pediatric urology, because of ethical and technical considerations and the significant small caseload and patient-related variability (3, 10).

The IDEAL collaboration is a framework that allows the assessment of a surgical technique or innovation according to the stages in which innovation in surgery occurs, from the development of the Idea, Development, Exploration, Assessment, and Long-term follow-up (11).

The objective of this work is to evaluate the technical development of HPBD in the treatment of patients with POM and its clinical outcome in a systematic way using the IDEAL framework as an analysis tool.

## Materials and Methods

A systematic search in Pub Med of “Primary Obstructive Megaureter” was conducted with a 10 years publication date filter (primary [All Fields] AND obstructive [All Fields] AND megaureter [All Fields]) AND (“2008/09/26”[PDat]: “2018/09/23”[PDat]).

A limit to 10-year literature review was set to focus in the most updated reported experience and matching the currently available technology and care standards, aiming to reduce variability, although acknowledging that this would dismiss some initial reports on the technique under research.

The criteria for entering the analysis of this surgical procedure using the IDEAL model as an assessment tool were “single technique on endoscopic HPBD and identifiable case series or cohorts.”

The PubMed search produced 69 entries (Diagram 1 in Supplementary Material).

Entries matching the analysis criteria (endoscopic high-pressure balloon dilatation patient series) were 10 (Annex 1 in Supplementary Material).

- One report corresponding to Garcia-Aparicio et al. was studied separately as it contained a comparative analysis with open ureteric reimplantation, and cohort data was included in a further publication from the same author (12).
- Seven cohorts from “High-Pressure Balloon Dilatation” HPBD were included for the analysis using the IDEAL framework and an analysis of the clinical outcomes.
- Two reports from Torino et al. and Garcia-Aparicio et al. were excluded from the analysis as the same cohort of patients were included in later publications included in the analysis (13, 14).

Other series or patient cohort identified were:

- Non-endoscopic series: 13 (Annex 2 in Supplementary Material)
- Endoscopic series: 6 (Annex 3 in Supplementary Material).

The reported experience in the papers included for the study were analyzed according to IDEAL framework to determine:

- Stage of the technique and its development
- Quality according to recommended criteria for designing and reporting studies of “surgical interventions”

The clinical data identifiable was also recorded and analyzed.

Stage 1, or Idea, represents “proof of concept” and usually describes first use of a technique or procedure in a human, prompted by the need of a solution for a clinical problem or challenging situation (7).

The development stage (2a) describes the technical refinement of a procedure through iterative modification and evaluation of short-term outcomes. The aim during this stage is to prove the stability of the technique and describe the safety, the technique, and both the technical and procedural success (8).

The exploration stage (2b) reports details of procedural quality and focuses on learning curves and compares the technique outcomes with the current gold standard (10). The aim would be to describe patient selection, indications, and results for the degree of success.

Usually, there would be uncontrolled cohort studies, from unconnected centers. In some occasions, there are publications from collaborating groups, registries, or collaborative audits.

Innovations that have reached stage 2b (exploration) usually have reports published from several unconnected centers. Most of these studies will be uncontrolled cohort studies, but there may also be reports from collaborative audits, registries, and other cooperative groups. Usually, numbers are more extensive than in stage 2a, typically including some reports with over 100 patients (10). However, this would be very unusual in the case of pediatric surgical specialities, as the caseload is usually very small compared to adult specialities, and there is a wide variety of the cases (15).

Stage 3 aims to compare against current accepted best practice or become a replacement to current techniques (7). Reports in this stage would characteristically show high-quality randomized control trial (RCT) or other valid experimental comparison of the intervention compared with the current treatment (11).

In stage 4 reflects long term monitoring and is demonstrated by prospective registries that should analyze both indications and outcomes (10).

Papers were also analyzed according to the recommended criteria for designing or reporting studies according to IDEAL (11):

- Transparency and completeness, reporting as “fully as possible” patient selection or exclusion criteria and follow up losses.
- Protocol and study registration, in this particular case focussed on the type of study, prospective/retrospective.
- Standardization of participants, confounders and outcomes reporting.

Seven identifiable cohorts were found and systematically analyzed using IDEAL proposed framework to identify the stage of the development of the technique and assess its quality and validity according to the key elements for each stage (Table 1) (11).

**Table 1:** IDEAL reported cohorts assessment.

**Stages**	**Publication**	**Characteristics and study design**	**Key issues contents**	**Transparency and completeness**	**Protocol and study registration**	**Standard definition participants/confounders/outcomes**
2a and 2b	Endoscopic balloon dilatation in primary obstructive megaureter: Long-term results (1).	Retrospective Observational 13 patients/121 POM	Short and long-term outcomes. Technique has reached stability and consensus	Eligibility + Patient selection + Recruitment period + FU methods + Diagnostic criteria + Quantitative variables and analysis +	No	Adequate cohort description + Standardized technique description− Standardized outcomes - Standardized FU+ Description of technical modifications − Description of learning curve −
2a and 2b	High pressure balloon dilatation of the ureterovesical junction in primary obstructive megaureter: Infectious morbidity (2).	Retrospective Observational 12 patients/12 units	Safety of the procedure. Short-term complications. Long term outcomes. Technique has reached stability and consensus	Eligibility − Patient selection + Recruitment period + FU methods + Diagnostic criteria + Quantitative variables and analysis +	Yes	Adequate cohort description + Standardized technique description− Standardized outcomes − Standardized FU+ Description of technical modifications − Description of learning curve −
2b	Postoperative vesicoureteral reflux after high-pressure balloon dilation of the ureterovesical junction in primary obstructive megaureter. Incidence, management, and predisposing factors (3).	Retrospective Observational 20 patients/22 units	Medium and long-term complications. Technnique has reached stability and consensus	Eligibility − Patient selection − Recruitment period + FU methods + Diagnostic criteria + Quantitative variables and analysis	No	Adequate cohort description + Standardized technique description− Standardized outcomes − Standardized FU+ Description of technical modifications − Description of learning curve −
2b	Can endoscopic balloon dilation for primary obstructive megaureter be effective in a long-term follow-up? (4).	Retrospective Observational 19 patients/20 ureters	Long-term outcomes Technique has reached stability and consensus	Eligibility + (non-specific data) Patient selection + Recruitment period + FU methods + Diagnostic criteria + Quantitative variables and analysis +	No	Adequate cohort description + Standardized technique description− Standardized outcomes + (Clavien) Standardized FU+ Description of technical modifications − Description of learning curve −
2b	Primary obstructive megaureter in infants: our experience with endoscopic balloon dilation and cutting balloon ureterotomy (5).	Retrospective/prospective? Observational HPBD 7/10 ureters		Eligibility + (non-specific data) Patient selection + Recruitment period + FU methods + Diagnostic criteria + Quantitative variables + and analysis −	No	Adequate cohort description + Standardized technique description− Standardized outcomes − Standardized FU+ Description of technical modifications − Description of learning curve −
2a and 2b	Primary Obstructive Megaureter: The Role of High Pressure Balloon Dilation (6).	Retrospective Observational 29 patients/32 units	Safety of the procedure Short-term and long-term outcomes Discusses procedural quality Discusses learning curve	Eligibility − Patient selection + Recruitment period + FU methods + Diagnostic criteria + Quantitative variables + and analysis −		Adequate cohort description + Standardized technique description + Standardized outcomes - Standardized FU+ Description of technical modifications + Description of learning curve +
2a and 2b	Endoscopic management and the role of double stenting for primary obstructive megaureters (7).	Retrospective/prospective? HPBD 12 cases	Safety of the procedure Short-term and long-term outcomes Discusses procedural quality and technical modifications	Eligibility − Patient selection + Recruitment period − FU methods + Diagnostic criteria + Quantitative variables and analysis	Yes	Adequate cohort description + Standardized technique description + Standardized outcomes − Standardized FU+ Description of technical modifications + Description of learning curve −

*POM, Primary obstructive megaureter; FU, Follow up; HPBD, High pressure balloon dilatation*.

## Results

The analysis of the results of the systematic assessment of the reported cohort of patients treated with HPBD for POM that the technique up to date is in stage 2a and stage 2b.

As described by Pennel et al. “differentiating stage 2a from 2b may be difficult from publications (11).” Moreover, I think this particularly applies to the techniques reviewed in this analysis, and this is also associated with the small size of the available cohorts.

The characteristics of the seven cohorts analyzed are in general, thoroughly reported but case selection and eligibility for “interventional treatment for POM” is not as systematic as recommended, except for the experience by Casal Beloy et al. (5).

Clinical outcomes and follow up are well-described in the seven reported cohorts, and they are consistent with the recommended criteria for retrospective observational studied proposed by the Template for Intervention Description and Replication (TIDieR) checklist (16).

Studies, although approved by local ethical committee's do not have a protocol registration before initiating enrolment with the associated risk of bias caused insufficient reporting of negative outcomes.

The reported cohorts are all analyzed retrospectively as well, that possess clinical findings under the threat of missing or inadequate data.

Data from adverse outcomes and complications are reported in all series analyzed, but systematization in reporting them using Clavien-Dindo classification is scarce (17).

Technical variations are well-illustrated, in line with the thorough description of the techniques and equipment and fungible used. Indication for these variations such as the use of a cutting balloon “second look or vesicoureteric junction (VUJ) assessment” (6) or exclusion criteria for HPBD are reported and explained in all of them (5, 18, 19).

Only one report (6) reflects the effect of the “learning curve” on the technical and clinical outcomes of HPBD in POM, and the rationale for variation in the technique based on this “learning curve.”

In the study by García-Aparicio et al. a comparative analysis of outcomes between ureteric reimplantation and HPBD for POM is done (12). This would represent a Stage 3 of the IDEAL framework, although it is also a retrospective study and lacks randomization and the number of cases analyzed is small.

Clinical outcomes of the seven identifiable cohorts were also analyzed for clinical results, and quality of data and results are shown in Table 2.

**Table 2:** Cohorts reported clinical outcomes.

**Publication**	**Cohort**	**Technical considerations**	**Operative complications**	**FU**	**Outcome**	**Successful**
Endoscopic balloon dilatation in primary obstructive megaureter: Long-term results (5).	121 POM 13 patients HPBD/13 ureters 5 patients open ureteric reimplantation	Distal ureter > 25 mm excluded from HPBD	No	Median 10.3 years (4.7–12.2 years)	Jj stent migration-replacement (*n =* 1) DRF stable (*n =* 12) improved (*n =* 1) Decrease in ureterohydronephrosis (*n =* 13) Ureteric reimplantation *n =* 0	12/12
High pressure balloon dilatation of the ureterovesical junction in primary obstructive megaureter: Infectious morbidity (20).	12 patients/12 ureters		No	Mean 26.5 months (20–44 months)	Redilation (*n =* 2) JJ stent related UTI (*n =* 7) [preoperative UTI (*n =* 4)] No UTIs after JJ stent removal DRF stable (*n =* 12) Ureteric reimplantation *n =* 1 (obstruction)	9/12
Postoperative vesicoureteral reflux after high-pressure balloon dilation of the ureterovesical junction in primary obstructive megaureter. Incidence, management and predisposing factors (12)	20 patients/22 ureters	VCUG routinely performed as FU protocol	No		Ureteric reimplantation *n =* 3 (obstruction) VUR *n =* 6 Ureteric reimplantation *n =* 2 (VUR) Endoscopic correction VUR *n =* 1	17/22
Can endoscopic balloon dilation for primary obstructive megaureter be effective in a long-term follow-up? (17).	19 patients/20 ureters	JJ stent insertion using an ureteral bridge	No Clavien I (*n =* ?)	Mean 6.9 years (3.9–13.3 years)	DFR stable *n =* 18 patients Redilation *n =* 1	19/20
Primary obstructive megaureter in infants: our experience with endoscopic balloon dilation and cutting balloon ureterotomy (18).	HPBD 7 patients/10 ureters	Technique based on intraoperative findings: • Ring and HPBD *n =* 7 • No ring *n =* 2 • Ring persisted after HPBD *n =* 3. CBD *n =* 3	No	Mean 21 months (2–44 months)	HPBD decrease ureterohydronephrosis 7/7 No ring at HPBD decrease of ureterohydronephrosis 2/2 Ureteric reimplantation 2/2 HPBD +CBD decrease ureterohydronephrosis 2/2 DRF stable 7/7 HPBD	5/7
Primary Obstructive Megaureter: the Role of High Pressure Balloon Dilation (6).	29 patients/32 ureters	Dilating catheter change (>5 mm) VUJ assessment at JJ stent removal	Yes Unable to perform procedure *n =* 2 Unable to insert JJ stent *n =* 2	Median 47 (IQR 39,07) months	Re-dilation *n =* 2 (dilating catheter changed upon this findings) Ureteric reimplantation HPBD not performed *n =* 2 Ureteric reimplantation *n =* 2 (Obstruction) Ureteric reimplantation *n =* 2 (VUR) Endoscopic correction of VUR *n =* 3 DRF stable 29/29 HPBD decrease ureterohydronephrosis *n =* 26	26/29
Endoscopic management and the role of double stenting for primary obstructive megaureters (19).	HPBD 12 cases	Exclusion “narrow segment” > 3 cm	No	Mean of 3.2 years (Range 2.0–6.5 years)	DRF (MRU) stable 12/12 MRU no obstruction 12/12 Decrease ureterohydronephrosis 7/12	12/12
Total cohorts	HPBD 114 units		4	2 months−13.3 years		100/114

*POM, Primary obstructive megaureter; FU, Follow up; VCUG, Voiding cystourethrogram; FU, Follow up; HPBD, High pressure balloon dilatation; CBD, Cutting balloon dilatation; DRF, Differential renal function; UTI, Urinary tract infection; VUR, Vesicoureteric reflux*.

The analysis of the reported clinical outcomes in this seven cohorts shows a success rate of HPBD of the vesicoureteral junction for POM of 87.7% for a follow-up period ranging from 2 months to 13.3 years. Complication rate reported was 3.5%.

From the comparative analysis of HPBD with open ureteric reimplantation (12), there were no significant differences in success rate or secondary vesicoureteral reflux requiring surgical repair.

The available literature analyzed in this study also shows that there is a small number of published works on surgical treatment for primary obstructive megaureter, only 29 papers in the last 10 years. Among them, there is not a single experience on what is believed to be the “state of the art” surgical approach for POM, open ureteric reimplantation with or without ureteric tapering, which may limit the available data for comparative analyzes, as this may mean that this is not as routinely performed or simply not reported anymore.

## Discussion

The role of endoscopic techniques in the management of children with POM that require surgical intervention remains controversial and clinical research about this topic has attracted criticism and reluctance. This has also been the case for HPBD of the vesicoureteral junction for POM.

The desirable, high-quality randomized trials to provide solid enough evidence on HPBD for POM may be an unrealistic expectation, as it is on many occasions in surgery. There are limitations in study design based in patient variability, clinician, and setting variability among other factors that may make it impossible.

Concern about the adoption of this or any other surgical innovation without adequate supporting evidence of efficacy and safety is always the case and affects research adversely. These phenomena may lead to slow adoption of innovations or rather the contrary, permit widespread adoption of procedures that offer no benefit, or cause harm.

Assessment of the quality of evidence is imperative, especially in areas that affect clinical decision making in pediatrics.

The Idea, Development, Exploration, Assessment, and Long-term Follow-up (IDEAL) Framework and Recommendations have been reported as an excellent tool to evaluate data or evidence quality in surgical research.

This study of the recently reported experience on HPBD for POM using IDEAL as a tool to assess evidence quality shows, that researchers have communicated their outcomes thoroughly. However, analyzed publications lack of a systematic outcome reporting that would definitely enhance the clinical decision-making process, and better clarify the role of HPBD in the management of POM.

Future research would benefit from reporting according to a standard set of measures for indication for surgery as retrovesical ureteral diameter, renal pelvis anteroposterior diameter, renogram parameters, and symptoms (21). Systematic outcome reporting of radiological and clinical outcomes, including postoperative clinical, radiological assessment, renographic parameters, and a definition of successful outcomes would also improve comparative analysis (2).

This IDEAL assessment may be the basis for future research in defining the “core outcome sets” for the interventional treatment of POM as recommended by the Core Outcome Measures in Effectiveness Trials Initiative.

Clinical outcomes are thoroughly reported as well as follow up, but other potential outcomes that may have a significant impact on the role of HPBD for POM are not even considered. Potential research outcomes such us cost-efficiency, patient/family experience or values are not analyzed, and therefore this missing information may have a substantial role in defining the appropriate comparative study among surgical interventions for POM.

The assessment shows that protocol registration and prospective data collection is required, not only for HPBD but for all other surgical interventions in POM.

An analysis of the non-HPBD series within the 10 year's study period demonstrates that data reporting quality is as limited or even more. Studying other historical series (1) that have been the foundations of the repeated recognition of ureteric reimplantation as “gold standard” for surgical management, they show poorer quality data than the analyzed HPBD series.

Although systematic reviews are a source of evidence, inadequate methodology assessing surgical interventions may lead to mistaken conclusions. This issue can be particularly crucial in POM because caseload is small and the temptation to analyze all the endoscopic techniques without an adequate tool may lead to erroneous conclusions (22).

Future research on HPBD for POM should be aimed to reach Stage 3 and gain consensus about systematization of technical details as much as appropriate indications and outcome measures. It will also require prospective data collection, both of patient-related clinical information and technology related data, other clinical outcomes (pain, time to recovery), patient/family experience, cost-efficiency.

Technical modifications proposed by authors should also undertake systematic assessment, such as better exclusion criteria (5–18) (diameter, not finding a stenotic segment), no JJ stent as morbidity associated with it is substantial.

The analysis using IDEAL as an assessment tool of this reported experience of the HPBD for POM in children demonstrates that the technique is stable, data quality is adequate.

The completion of this stage would be the basis for a more definitive study comparing the new procedure with other surgical approaches.

Clinical outcomes of HPBD in the management of POM consistently show a success rate >87% with a negligible operative complication rate once “learning curve” has been surpassed.

Symptomatic vesicoureteral reflux is the main reason for ureteric reimplantation, but asymptomatic VUR does not seem to influence clinical outcome.

Comparative analysis with classical approaches or other endoscopic or minimally invasive techniques requires a consensus on the set of outcomes and an improvement in research quality.

The IDEAL framework and recommendations are excellent tools in designing future research that would assist clinicians now and in the future to improve patient management.

## Author Contributions

The author confirms being the sole contributor of this work and has approved it for publication.

### Conflict of Interest Statement

The author declares that the research was conducted in the absence of any commercial or financial relationships that could be construed as a potential conflict of interest. The handling Editor declared a past co-authorship with the author.
