# Supplementary material for: Management of Primary Obstructive Megaureter by Endoscopic High-Pressure Balloon Dilatation. IDEAL Framework Model as a New Tool for Systematic Review
Source: Front Surg. 2019 Apr 16;6:20. doi: 10.3389/fsurg.2019.00020 (PMC6478015; doi:10.3389/fsurg.2019.00020)
Supplement: Supplementary file 1 [file Data_Sheet_1.docx]

Annex 1

1: Casal Beloy I, Somoza Argibay I, García González M, García Novoa MA, Míguez

Fortes LM, Dargallo Carbonell T. Endoscopic balloon dilatation in primary

obstructive megaureter: Long-term results. J Pediatr Urol. 2018

Apr;14(2):167.e1-167.e5. doi: 10.1016/j.jpurol.2017.10.016. Epub 2017 Nov 21.

PubMed PMID: 29398584.

2: Kassite I, Braïk K, Morel B, Villemagne T, Szwarc C, Maakaroun Z, Cook AR,

Lardy H, Binet A. High pressure balloon dilatation of the ureterovesical junction

in primary obstructive megaureter: Infectious morbidity. Prog Urol. 2017

Sep;27(10):507-512. doi: 10.1016/j.purol.2017.07.005. Epub 2017 Sep 1. PubMed

PMID: 28867581.

3: García-Aparicio L, Blázquez-Gómez E, de Haro I, Garcia-Smith N, Bejarano M,

Martin O, Rodo J. Postoperative vesicoureteral reflux after high-pressure balloon

dilation of the ureterovesical junction in primary obstructive megaureter.

Incidence, management and predisposing factors. World J Urol. 2015

Dec;33(12):2103-6. doi: 10.1007/s00345-015-1565-9. Epub 2015 Apr 22. PubMed PMID:

25899625.

4: Bujons A, Saldaña L, Caffaratti J, Garat JM, Angerri O, Villavicencio H. Can

endoscopic balloon dilation for primary obstructive megaureter be effective in a

long-term follow-up? J Pediatr Urol. 2015 Feb;11(1):37.e1-6. doi:

10.1016/j.jpurol.2014.09.005. Epub 2015 Feb 7. PubMed PMID: 25748631.

5: Capozza N, Torino G, Nappo S, Collura G, Mele E. Primary obstructive

megaureter in infants: our experience with endoscopic balloon dilation and

cutting balloon ureterotomy. J Endourol. 2015 Jan;29(1):1-5. doi:

10.1089/end.2013.0665. PubMed PMID: 24646018.

6: Romero RM, Angulo JM, Parente A, Rivas S, Tardáguila AR. Primary obstructive

megaureter: the role of high pressure balloon dilation. J Endourol. 2014

May;28(5):517-23. doi: 10.1089/end.2013.0210. Epub 2014 Feb 14. PubMed PMID:

24400855.

~~7: García-Aparicio L, Blázquez-Gómez E, Martin O, Palazón P, Manzanares A,~~

~~García-Smith N, Bejarano M, de Haro I, Ribó JM. Use of high-pressure balloon~~

~~dilatation of the ureterovesical junction instead of ureteral reimplantation to~~

~~treat primary obstructive megaureter: is it justified? J Pediatr Urol. 2013~~

~~Dec;9(6 Pt B):1229-33. doi: 10.1016/j.jpurol.2013.05.019. Epub 2013 Jun 21.~~

~~PubMed PMID: 23796389.~~

~~8: García-Aparicio L, Rodo J, Krauel L, Palazon P, Martin O, Ribó JM. High~~

~~pressure balloon dilation of the ureterovesical junction--first line approach to~~

~~treat primary obstructive megaureter? J Urol. 2012 May;187(5):1834-8. doi:~~

~~10.1016/j.juro.2011.12.098. Epub 2012 Mar 16. PubMed PMID: 22425047.~~

9: Christman MS, Kasturi S, Lambert SM, Kovell RC, Casale P. Endoscopic

management and the role of double stenting for primary obstructive megaureters. J

Urol. 2012 Mar;187(3):1018-22. doi: 10.1016/j.juro.2011.10.168. Epub 2012 Jan 20.

PubMed PMID: 22264463.

~~10: Torino G, Collura G, Mele E, Garganese MC, Capozza N. Severe primary~~

~~obstructive megaureter in the first year of life: preliminary experience with~~

~~endoscopic balloon dilation. J Endourol. 2012 Apr;26(4):325-9. doi:~~

~~10.1089/end.2011.0399. Epub 2011 Dec 7. PubMed PMID: 22050492.~~
