# Supplementary material for: Management of Primary Obstructive Megaureter by Endoscopic High-Pressure Balloon Dilatation. IDEAL Framework Model as a New Tool for Systematic Review
Source: Front Surg. 2019 Apr 16;6:20. doi: 10.3389/fsurg.2019.00020 (PMC6478015; doi:10.3389/fsurg.2019.00020)
Supplement: Supplementary file 2 [file Data_Sheet_2.docx]

Annex 2

1: Shrestha AL, Bal HS, Kisku SMC, Sen S. Outcome of end cutaneous ureterostomy

(ECU) as a non conservative option in the management of primary obstructive

megaureters (POM). J Pediatr Urol. 2018 Jun 7. pii: S1477-5131(18)30235-3. doi:

10.1016/j.jpurol.2018.05.004. [Epub ahead of print] PubMed PMID: 29937413.

2: Lopez M, Gander R, Royo G, Varlet F, Asensio M. Laparoscopic-Assisted

Extravesical Ureteral Reimplantation and Extracorporeal Ureteral Tapering Repair

for Primary Obstructive Megaureter in Children. J Laparoendosc Adv Surg Tech A.

2017 Aug;27(8):851-857. doi: 10.1089/lap.2016.0456. Epub 2017 Jun 8. PubMed PMID:

28594594.

3: Cardoso Braz P, Ghilardi Leão F, Quintino de Souza Leão J, Silveira Onofre L,

Machado Campos de Oliveira G, Carnevale J, Miguélez Lago C. Ureteral meatotomy as

an option in the management of primary obstructive megaureter in neonates and

infants. Arch Esp Urol. 2017 Apr;70(3):349-356. Spanish, English. PubMed PMID:

28422037.

4: Khan A, Rahiman M, Verma A, Bhargava R. Novel technique of laparoscopic

extravesical ureteric reimplantation in primary obstructive megaureter. Urol Ann.

2017 Apr-Jun;9(2):150-152. doi: 10.4103/0974-7796.204182. PubMed PMID: 28479766;

PubMed Central PMCID: PMC5405658.

5: Liu W, Du G, Guo F, Ma R, Wu R. Modified ureteral orthotopic reimplantation

method for managing infant primary obstructive megaureter: a preliminary study.

Int Urol Nephrol. 2016 Dec;48(12):1937-1941. Epub 2016 Sep 2. PubMed PMID:

27590133.

6: Abou Youssif TM, Fahmy A, Rashad H, Atta MA. The embedded nipple: An optimal

technique for re-implantation of primary obstructed megaureter in children. Arab

J Urol. 2016 May 20;14(2):171-7. doi: 10.1016/j.aju.2016.04.001. eCollection 2016

Jun. PubMed PMID: 27493810; PubMed Central PMCID: PMC4963169.

7: Gundeti MS, Boysen WR, Shah A. Robot-assisted Laparoscopic Extravesical

Ureteral Reimplantation: Technique Modifications Contribute to Optimized

Outcomes. Eur Urol. 2016 Nov;70(5):818-823. doi: 10.1016/j.eururo.2016.02.065.

Epub 2016 Mar 30. PubMed PMID: 27036858.

8: Liu X, Liu JH, Zhang DY, Hua Y, Lin T, Wei GH, He DW. Retrospective study to

determine the short-term outcomes of a modified pneumovesical Glenn-Anderson

procedure for treating primary obstructing megaureter. J Pediatr Urol. 2015

Oct;11(5):266.e1-6. doi: 10.1016/j.jpurol.2015.03.020. Epub 2015 Jun 4. PubMed

PMID: 26076822.

9: Chandrasekharam VV. Laparoscopic undiversion of end ureterostomy: A novel

technique. J Pediatr Urol. 2015 Jun;11(3):161-3. doi:

10.1016/j.jpurol.2015.03.005. Epub 2015 Apr 23. PubMed PMID: 25964197.

10: García-Aparicio L, Blázquez-Gómez E, Martin O, Palazón P, Manzanares A,

García-Smith N, Bejarano M, de Haro I, Ribó JM. Use of high-pressure balloon

dilatation of the ureterovesical junction instead of ureteral reimplantation to

treat primary obstructive megaureter: is it justified? J Pediatr Urol. 2013

Dec;9(6 Pt B):1229-33. doi: 10.1016/j.jpurol.2013.05.019. Epub 2013 Jun 21.

PubMed PMID: 23796389.

11: Bondarenko S. Laparoscopic extravesical transverse ureteral reimplantation in

children with obstructive megaureter. J Pediatr Urol. 2013 Aug;9(4):437-41. doi:

10.1016/j.jpurol.2013.01.001. Epub 2013 Mar 11. PubMed PMID: 23491982.

12: He Y, Chen X, Chen Z, Luo YC, Li NN. Treatment of symptomatic primary

obstructive megaureter by laparoscopic intracorporeal or extracorporeal ureteral

tapering and ureteroneocystostomy: experience on 11 patients. J Endourol. 2012

Nov;26(11):1454-7. doi: 10.1089/end.2012.0236. Epub 2012 Oct 16. PubMed PMID:

22691071.

13: Mitre AI, Lestingi JF, Arap MA, Lucon AM, Srougi M. Totally laparoscopic

ureteroneocystostomy with intracorporeal tailoring for primary obstructive

megaureter. Clinics (Sao Paulo). 2011;66(1):177-9. PubMed PMID: 21437458; PubMed

Central PMCID: PMC3044588.

14: Hemal AK, Nayyar R, Rao R. Robotic repair of primary symptomatic obstructive

megaureter with intracorporeal or extracorporeal ureteric tapering and

ureteroneocystostomy. J Endourol. 2009 Dec;23(12):2041-6. doi:

10.1089/end.2009.0103. PubMed PMID: 19909072.
