# Supplementary material for: Management of Primary Obstructive Megaureter by Endoscopic High-Pressure Balloon Dilatation. IDEAL Framework Model as a New Tool for Systematic Review
Source: Front Surg. 2019 Apr 16;6:20. doi: 10.3389/fsurg.2019.00020 (PMC6478015; doi:10.3389/fsurg.2019.00020)
Supplement: Supplementary file 3 [file Data_Sheet_3.docx]

Annex 3

1: Teklali Y, Robert Y, Boillot B, Overs C, Piolat C, Rabattu PY. Endoscopic

management of primary obstructive megaureter in pediatrics. J Pediatr Urol. 2018

Jun 28. pii: S1477-5131(18)30323-1. doi: 10.1016/j.jpurol.2018.05.027. [Epub

ahead of print] Review. PubMed PMID: 30006257.

2: Shirazi M, Natami M, Hekmati P, Farsiani M. Result of endoureterotomy in the

management of primary obstructive megaureter in the first year of life:

preliminary report. J Endourol. 2014 Jan;28(1):79-83. doi: 10.1089/end.2013.0098.

Epub 2013 Oct 17. PubMed PMID: 23937376.

3: Smeulders N, Yankovic F, Chippington S, Cherian A. Primary obstructive

megaureter: cutting balloon endo-ureterotomy. J Pediatr Urol. 2013

Oct;9(5):692.e1-2. doi: 10.1016/j.jpurol.2013.04.010. Epub 2013 Jun 5. PubMed

PMID: 23759477.

4: Kajbafzadeh AM, Tourchi A. Concomitant endoureterotomy and

dextranomer/hyaluronic acid subureteral injection for management of obstructive

refluxing megaureter. J Endourol. 2012 Apr;26(4):318-24. doi:

10.1089/end.2011.0256. PubMed PMID: 22059739.

5: Carroll D, Chandran H, Joshi A, McCarthy LS, Parashar K. Endoscopic placement

of double-J ureteric stents in children as a treatment for primary obstructive

megaureter. Urol Ann. 2010 Sep;2(3):114-8. doi: 10.4103/0974-7796.68860. PubMed

PMID: 20981199; PubMed Central PMCID: PMC2955226.

6: Farrugia MK, Steinbrecher HA, Malone PS. The utilization of stents in the

management of primary obstructive megaureters requiring intervention before 1

year of age. J Pediatr Urol. 2011 Apr;7(2):198-202. doi:

10.1016/j.jpurol.2010.04.015. Epub 2010 May 21. PubMed PMID: 20494618.
