# Supplementary material for: Management of Primary Obstructive Megaureter by Endoscopic High-Pressure Balloon Dilatation. IDEAL Framework Model as a New Tool for Systematic Review
Source: Front Surg. 2019 Apr 16;6:20. doi: 10.3389/fsurg.2019.00020 (PMC6478015; doi:10.3389/fsurg.2019.00020)
Supplement: Supplementary file 4 [file Data_Sheet_4.docx]

“Primary Obstructive Megaureter”

(2008/09/26 - 2018/09/23)

n= 69

Other endoscopic series

n= 13

High pressure balloon dilatation

Identifiable cohorts

n= 10

Non-endoscopic series

n= 13

Excluded

n=3

Analysis

N=7
